# Determining Best Practices for Management of Bacteriuria in Spinal Cord Injury: Protocol for a Mixed-Methods Study

**DOI:** 10.2196/12272

**Published:** 2019-02-14

**Authors:** Felicia Skelton, Lindsey Ann Martin, Charlesnika T Evans, Jennifer Kramer, Larissa Grigoryan, Peter Richardson, Mark E Kunik, Ivy Oiyee Poon, S Ann Holmes, Barbara W Trautner

**Affiliations:** 1 Center for Innovations in Quality, Effectiveness and Safety Houston, TX United States; 2 H Ben Taub Department of Physical Medicine and Rehabilitation Baylor College of Medicine Houston, TX United States; 3 Health Services Research Section Department of Medicine Baylor College of Medicine Houston, TX United States; 4 Center for Innovation for Complex Chronic HealthCare Hines VA Hospital Chicago, IL United States; 5 Department of Preventive Medicine and Center for Health Care Studies Feinberg School of Medicine Northwestern University Chicago, IL United States; 6 Department of Family and Community Medicine Baylor College of Medicine Houston, TX United States; 7 Menninger Department of Psychiatry and Behavioral Sciences Baylor College of Medicine Houston, TX United States; 8 Department of Pharmacy Practice Texas Southern University Houston, TX United States; 9 Infectious Disease Section Department of Medicine Baylor College of Medicine Houston, TX United States

**Keywords:** spinal cord injury, urinary tract infection, patient-focused care, qualitative evaluation, antimicrobial stewardship

## Abstract

**Background:**

Bacteriuria, either asymptomatic (ASB) or symptomatic, urinary tract infection (UTI), is common in persons with spinal cord injury (SCI). Current Veterans Health Administration (VHA) guidelines recommend a screening urinalysis and urine culture for every veteran with SCI during annual evaluation, even when asymptomatic, which is contrary to other national guidelines. Our preliminary data suggest that a positive urine culture (even without signs or symptoms of infection) drives antibiotic use.

**Objective:**

Through a series of innovative studies utilizing mixed methods, administrative databases, and focus groups, we will gain further knowledge about the attitudes driving current urine testing practices during the annual exam, as well as quantitative data on the clinical outcomes of these practices.

**Methods:**

Aim 1 will identify patient, provider, and facility factors driving bacteriuria testing and subsequent antibiotic use after the SCI annual evaluation through qualitative interviews and quantitative surveys. Aim 2 will use national VHA databases to identify the predictors of urine testing and subsequent antibiotic use during the annual examination and compare the clinical outcomes of those who received antibiotics with those who did not. Aim 3 will use the information gathered from the previous 2 aims to develop the *Test Smart, Treat Smart* intervention, a combination of patient and provider education and resources that will help stakeholders have informed conversations about urine testing and antibiotic use; feasibility will be tested at a single site.

**Results:**

This protocol received institutional review board and VHA Research and Development approval in July 2017, and Veterans Affairs Health Services Research and Development funding started on November 2017. As of submission of this manuscript, 10/15 (67%) of the target goal of provider interviews were complete, and 77/100 (77%) of the goal of surveys. With regard to patients, 5/15 (33%) of the target goal of interviews were complete, and 20/100 (20%) of the target goal of surveys had been completed. Preliminary analyses are ongoing; the study team plans to present these results in April 2019. Database analyses for aim 2 will begin in January 2019.

**Conclusions:**

The negative consequences of antibiotic overuse and antibiotic resistance are well-documented and have national and even global implications. This study will develop an intervention aimed to educate stakeholders on evidence-based management of ASB and UTI and guide antibiotic stewardship in this high-risk population. The next step will be to refine the intervention and test its feasibility and effectiveness at multiple sites as well as reform policy for management of this common but burdensome condition.

**International Registered Report Identifier (IRRID):**

DERR1-10.2196/12272

## Introduction

### Background

Antibiotic stewardship (promoting appropriate use of antibiotics) is a high-level policy and public health initiative, as shown by recent mandates from the Center for Disease Control and the United Nations [1,2]. Persons with spinal cord injury (SCI) are vulnerable to inappropriate antibiotic use because of their medical complexity and frequent health care contact [3]. Specifically, bacteriuria is a common consequence of neurogenic bladder after SCI; asymptomatic bacteriuria (ASB) has a prevalence of 30% to 90% depending on the bladder management strategy used [4]. For comparison, the prevalence rate in healthy premenopausal women is 5%. ASB, defined as the presence of bacteria in the urine of a person not otherwise having signs or symptoms of a urinary tract infection (UTI), does not require treatment except in pregnancy and before urologic procedures [4]. In fact, evidence-based guidelines published by the Infectious Diseases Society of America recommend against collecting screening urine cultures or treating ASB in persons with SCI [4,5]. The Veterans Health Administration (VHA) guidelines outlining care for persons with SCI, however, recommend a yearly urinalysis and urine culture as part of an annual physical checkup, regardless of whether signs or symptoms of infection are present [6]. Obtaining these tests in asymptomatic patients is essentially a screening for ASB. Although the VHA guideline does not explicitly recommend treatment of ASB, review of 2 years of annual examination visits uncovered that 35% of cases of ASB were subsequently treated with antibiotics [7].

Our goal is to develop an effective antibiotic stewardship program for bacteriuria tailored to the SCI population and SCI providers. To address gaps in knowledge relevant to antibiotic stewardship in SCI, we will utilize a mixed methods approach. First, we will answer the following question: do providers and persons with SCI believe that testing the urine and treating asymptomatic colonization will lead to better outcomes? We will explore this by conducting qualitative interviews with patients with SCI and SCI providers to understand their perceptions and expectations related to having their urine tested annually, and being prescribed and adhering to prescribed antibiotics, and correlating this information with quantitative knowledge surveys. Next, we hypothesize that urine testing during the annual examination leads to antibiotic use, and the antibiotics, in turn, have downstream consequences. This will be examined through analysis of VHA data sources. Finally, we will develop an intervention to more effectively deliver evidence-based bacteriuria management to persons with SCI. We hypothesized that the intervention will be feasible to use and increase patient satisfaction with bacteriuria management during the annual evaluation.

### Conceptual Framework

Cabana et al and others have explored the barriers to successful implementation of clinical practice guidelines into actual practice [8]. Clinical practice guidelines for bacteriuria management are often long and complex, requiring users to keep a sequential mental record of the statements to arrive at the diagnosis of UTI or ASB. Clinical practice guidelines may conflict with the users’ pre-existing biases of the standard of care, also limiting their use [9,10]. For example, many providers that take care of persons with SCI believe that treating ASB from urease-producing organisms such as *Proteus* species is beneficial, but the evidence is not convincing toward this [11,12]. Our project will utilize the Cabana framework to lessen the diagnostic challenge of distinguishing UTI from ASB.

## Methods

### Project Design Overview

**Figure 1:**
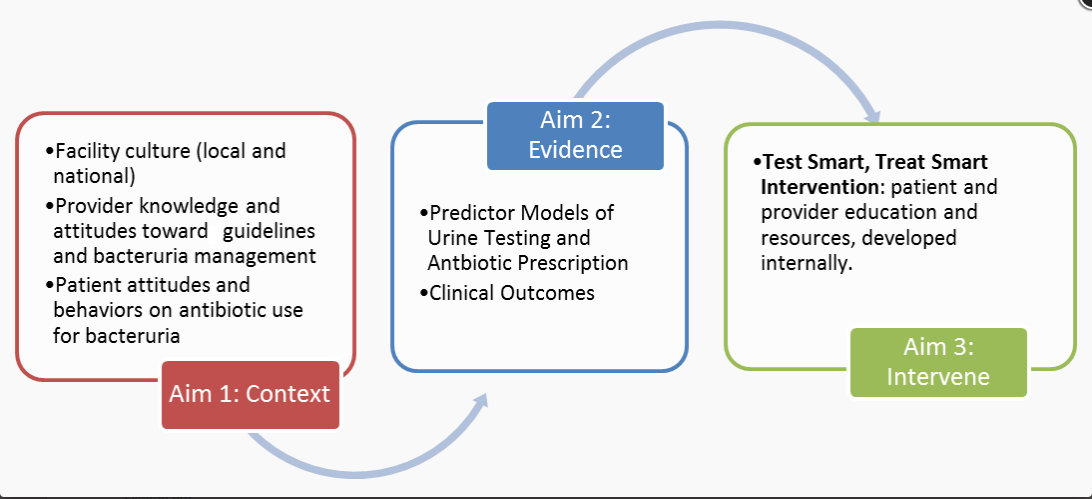
Optimizing bacteriuria management in the veteran spinal cord injury population. An overview of the planned project.

Figure 1 provides an overview of the project aims. We will identify *contextual factors* influencing provider and patient knowledge and beliefs about urine testing and treatment at the annual examination, using quantitative and qualitative methods (aim 1). We will then identify the *evidence* regarding patient, provider, and facility predictors of urine testing and subsequent antibiotic use, as well as compare the clinical outcomes of those who received antibiotics with the outcomes of those who did not (aim 2). We will then use information gained from the above aims and previously successful antibiotic stewardship initiatives [5] to *intervene*, by providing evidence-based bacteriuria management through education and resources for patients and providers (aim 3).

To develop an effective intervention to lessen the burden of utilizing clinical practice guidelines, we need a complete understanding of the key factors that drive provider testing and treatment behaviors (eg, knowledge and attitudes) and patient engagement. We will utilize the Cabana model, as described above, to understand provider barriers to using clinical practice guidelines in clinical practice [8]. During the development of the intervention (aim 3), we will use the concept of intervention mapping described by Kok et al [13], as well as audit and feedback, as a main component of the intervention. Intervention mapping is a process to develop theory- and evidence-based interventions and involves the following 6 steps: (1) needs assessment; (2) identification of change objectives; (3) selection of theory-based intervention methods and practical applications to enact change; (4) development of intervention components; (5) intervention adoption, implementation, and maintenance plan; and (6) plan for evaluation of intervention effectiveness. Audit and feedback or providing health care professionals with up-to-date data about their performance, has previously been shown to improve quality of care and was a successful component of the *Kicking catheter-associated urinary tract infection (CAUTI)* intervention [14,15]. We have also included a medical anthropologist in our team to explore how organizational culture intersects with and shapes medical practices in SCI clinics.

### Aim 1

This aim focuses on identifying patient, provider, and facility factors driving bacteriuria management in persons with SCI. We will employ qualitative and quantitative phases to identify patient, provider, and facility factors driving urine testing and antibiotic use during the annual evaluation.

#### Participants and Approach: Qualitative Phase

Semistructured, open-ended interviews will be conducted with health care providers (n=15) at 5 national VHA SCI outpatient centers (ie, hub sites) and 2 to 3 of their corresponding satellite primary care clinics (ie, spoke sites) and SCI patients (n=15). Maximum variation sampling, a purposeful sampling strategy, will guide our approach [16]. We are deliberately including physicians and nonphysician (ie, physician assistants and nurse practitioners) providers to capture a broad variety of experiences, and identify overarching themes, across geographically diverse sites; this sample size will allow for maximum variation in our data [17]. Our patient sample will be inclusive of factors such as age, ethnicity or race, and bladder management strategy (ie, indwelling catheter, intermittent catheter, etc) to capture a wide range of perspectives.

The interview guides are based on the major domains of our conceptual model. Provider interviews will begin by collecting demographic information that will include age, level of training, and information on board certification (physical medicine and rehabilitation, SCI medicine board-certified, and/or other specialties). Additional provider questions will focus on the utility of obtaining an annual urinalysis or urine culture and attitudes toward antibiotic stewardship. Patient interviews will begin by collecting demographic information that includes age, level of injury, numbers of years since injury, and method of bladder management. Patient interviews will focus on related topics discussed during the provider interviews, as well as previous experiences with UTI treatment and medication adherence.

#### Participants and Approach: Quantitative Phase

Following the interviews, all SCI patients and providers will be invited to participate in phone surveys. Patients who received a prescription for antibiotics for UTI will be invited to complete the Morisky Medication Adherence Scale-8 (MMAS-8). This is an 8-item validated questionnaire to evaluate intentional and unintentional medication nonadherence [18-20]. Providers will receive a closed-ended survey to assess knowledge in 6 domains about bacteriuria management. This is a validated survey used previously to explore this topic [21].

#### Analysis for Aim 1

Analysis of the qualitative and quantitative data will be concurrent. For the qualitative analysis, a combined inductive and deductive coding approach will be used to code the data. Moreover, 2 members of the research team (FS and LM) will read through the transcripts and develop a list of codes, based on participants’ experiences (inductive) and the subelements of our conceptual model (deductive). The full research team will hold regular meetings to discuss results and resolve any discrepancies in the coding process. Once coding is complete, individual codes will be sorted (ie, grouped together into like categories) by the research team to identify larger themes [22]. Qualitative analysis software, Atlas.ti, (Scientific Software Development GmbH) will be utilized to facilitate the coding process.

For the quantitative analysis, the patient scores on the MMAS-8 will be reported using descriptive statistics (mean, median, interquartile range, etc). The provider surveys provide data in the form of self-report ASB/UTI guidelines familiarity score, a knowledge score (series of hypothetical clinical scenarios testing the application of ASB/UTI guidelines), and a cognitive-behavioral domain score. We have previously used this survey to assess knowledge and behavior concerning ASB in acute and long-term care [21]. The knowledge score is the percentage of correct answers to the hypothetical clinical scenarios, with each correct response receiving 1 point. Descriptive statistics will be used to report the mean knowledge scores for various groups of respondents, and analysis of variance will be used to compare knowledge scores by provider type (ie, attending physician, physician assistant, and nurse practitioner, etc) and level of training. The relationship between the guideline familiarity score and knowledge score will be assessed using a Pearson correlation coefficient. The cognitive behavioral responses in each domain will be averaged to generate a numerical score for that domain. Correlations between the cognitive behavioral constructs and knowledge score will be calculated using the Pearson correlation coefficient.

The quantitative and qualitative data for patient and providers will then be integrated. For patients, we will integrate the mean scores on the MMAS-8 to interview responses on adherence to antibiotics. For providers, we will integrate the knowledge and guideline familiarity scores from the survey with their interview responses on familiarity with the guidelines. Congruence and incongruence between the quantitative and qualitative findings will be explored; for example, if providers answered in the affirmative about knowledge of guidelines, we will correlate the qualitative data with their scores on the knowledge survey.

### Aim 2

This objective will determine (1) which patient, provider, and facility factors are predictors of urine testing and subsequent antibiotic use during the annual evaluation and (2) compare the clinical outcomes of those who received antibiotics with the outcomes of those who did not, utilizing national VHA data sources. These databases (especially for use in SCI) have been described previously [23,24]. The corporate data warehouse (CDW) is a national repository including clinical and administrative data from the VHA. Data are stored in a relational database and are updated on a continual basis. We will use data from several domains within CDW to obtain demographics on the patient population (age, gender, race or ethnicity, and marital status); diagnoses, individual patient utilization (number of visits and admissions), provider type, and facility characteristics (SCI center vs non-SCI center and number of visits and admissions for the facility); temperature and heart rate; and laboratory data including albumin, creatinine, total white blood cell count, urea, glucose, hematocrit, electrolyte panels, and microbiology data (ie, date and time of culture, specimen type, organisms, and antibiotic susceptibilities). Outpatient medications to assess antibiotics filled and history of exposure to antibiotics will be obtained from all available outpatient pharmacy domains.

#### Participants

All adult patients with SCI treated at VHA facilities for an outpatient annual examination during the years 2015 and 2016 will be included. The study sample will be drawn from a cumulative list of veterans with SCI maintained by the Veterans Affairs Allocation Resource Center since 1988, which includes approximately 33,000 patients. Veterans are added to the list when an administrative record indicates an SCI in the inpatient diagnostic field with certain International Classification of Disease (ICD)-10 code.

The inclusion criteria for this aim are veterans who were seen for their annual examination in the outpatient setting in the VHA SCI system of care during the years 2015 and 2016 (approximately 6300 veterans are seen each year across the nation). Exclusion criteria are veterans who were seen for their SCI annual evaluation in the inpatient setting because visits may be complicated by other acute medical issues occurring at the time. The study will also exclude veterans with a history of genitourinary tract tumors per ICD-10 codes (and, therefore, more likely to have altered anatomy and/or immunosuppression), as well as those that died less than a year from the annual evaluation encounter. Only the first eligible encounter for each participant will be included. On the basis of our study looking at bacteriuria management in a single VHA center [7], we anticipate approximately 6000 participants after applying the exclusion criteria.

#### Approach

Aim 2a is to determine which patient, provider, and facility factors are predictors of urine testing and subsequent antibiotic use. To identify the annual examination encounter, we will use outpatient visits in the clinic stop code for SCI (210) that also has a current procedural terminology code for a renal ultrasound (usually ordered only during the annual evaluation).

**Figure 2:**
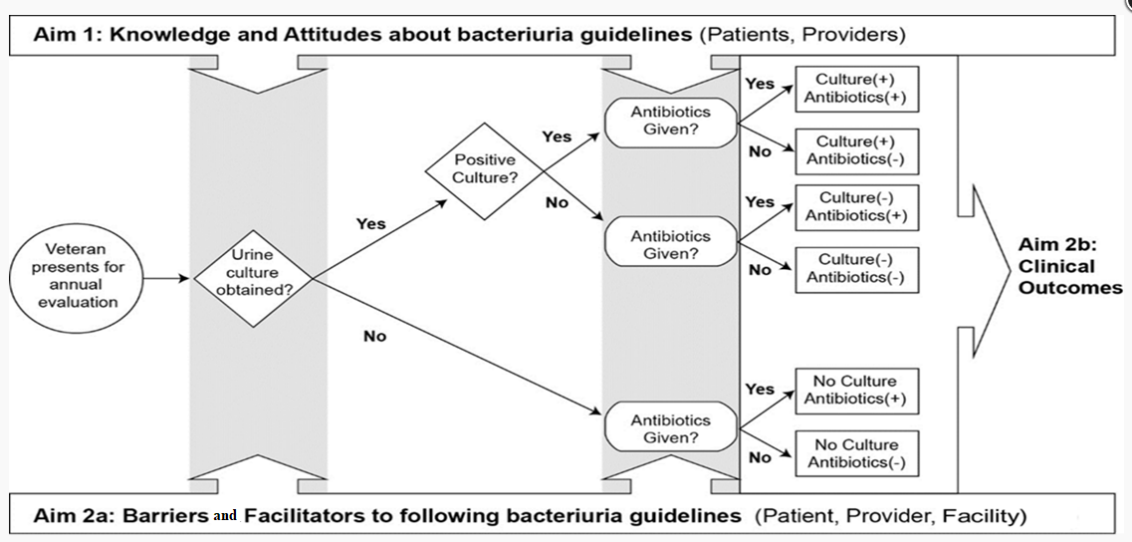
Bacteriuria management decision making in the veteran spinal cord injury population.

The general schema for this objective is shown in Figure 2. This is derived from the Cabana model for adherence to clinical guidelines.

Themes from aim 1 will guide an administrative database search of clinical outcomes of the current VHA urine testing and treatment practices.

The variables to be collected are described in Table 1. These were generated by the preliminary study results [7] and will be amended, based on other themes identified from objective 1. Zip codes of addresses will be obtained and given a GeoScore, a type of neighborhood socioeconomic index that is emerging as health services and health disparity research and is moving toward large administrative datasets, and was used by Hamilton et al exploring socioeconomic impact on health care utilization after SCI [25]. Deyo comorbidity index will also be calculated using VHA data sources.

Aim 2b will compare the clinical outcomes of those who received antibiotics with the outcomes of those who did not. We will use the same cohort as defined above. Table 2 defines the exposure and outcome variables, as well as covariates that will be considered for the second part of this aim.

#### Analysis for Aim 2

It would be clinically meaningful to show no difference in the outcomes between the 2 groups as we hypothesized that one driver of treating ASB at the annual exam is to prevent some of these negative downstream effects.

We will construct separate logistic regression models to determine the predictors of the following: (1) urine testing and (2) the initiation of antibiotics in the 7 days following the annual examination, with day 1 being the day of the examination. First, univariate analyses will be performed between each exposure variable (Table 1) and the outcome variables. Variables that satisfy a previously established *P* value criterion (.25) on univariate analysis will be considered for entry into a multivariate logistic regression model. We will use a lower *P* value threshold for the univariate analysis to ensure a robust model. Odds ratios will be used to determine the impact of each variable on antibiotic use. The alpha of .05 will be used to determine statistical significance. Multivariable logistic regression will be used to test all variables for independent associations. For aim 2b, rates of the above outcomes between those who received antibiotics versus those who did not will be compared using chi-square tests for nominal variables and *t* tests for continuous variables. The Mann-Whitney nonparametric U test will be used if the distribution of any continuous variable is not normal. We will construct a logistic regression model to determine the effect of antibiotics in the 7 days following the annual examination on the outcomes defined in Table 2. Univariate and multivariate analyses will be the same as aim 2a.

### Aim 3

The provider components of the *Test Smart, Treat Smart* intervention (aim 3a) will be an adaptation of the successful *Kicking CAUTI* intervention, consisting of audit and feedback, visual aids, and order sets, refined for SCI providers using iterative design. The patient components will be developed more de novo but be driven by previous work on how patients with SCI prefer to receive information (combination of internet and provider-driven sources) as well as the concept of intervention mapping described above [13,26,27].

Aims 3a and 3b will complete steps 1 to 4 of the intervention mapping process and lay the foundation for step 5 by pilot testing the intervention to assess feasibility (aim 3c). Specifically, we will assess time to complete the intervention, perceived burden to providers, and patient satisfaction.

**Table 1:** Definition of variables for aim 2a.

Variable type and name			Definition
**Exposure variables**			
	**Patient factors**		
		Age at encounter	Calculated using date of birth CDW^a^ patient domain
		Gender	Male or female
		Ethnicity or race	As per VHA^b^ convention
		Socioeconomic status	GeoScore to zip code to median income
		Neurologic level of injury	ICD-10^c^ codes for complete and incomplete quadriplegia and paraplegia
		Bladder management strategy	Catheter prescriptions in pharmacy data
		Urease-producing organism on culture	Determine positive culture from CDW Micro
		Level of pyuria on urinalysis	Use as continuous variable from CDW Micro
		Number of clinic visits per year	Use clinic stop code 210 for SCI^d^ clinic
		Others (as determined by aim 1)	—^e^
	**Provider factors**		
		Provider type	Physician, physician assistant
		Provider load of SCI patients	Number of SCI patients seen by provider in 2015 and 2016
		Others (as determined by Aim 1)	—
	**Facility factors**		
		Seen at SCI center (hub) or SCI satellite clinic (spoke)	Identify by unique facility codes from majority of visits
		Geographical region	Using VA^f^ Citrix Access Groupings for north, south, east, and west
		Facility complexity	General knowledge code that is applied to each facility
		Presence of antimicrobial stewardship program	Provide results from Healthcare Analysis and Information Group (HAIG) 2012 survey
		Others (as determined by aim 1)	—
**Outcome variables**			
		Urine culture obtained	Identified in CDW microdomain
		Antibiotics given for urine within 7 days	Antibiotic prescription noted in CDW outpatient pharmacy domain

^a^CDW: corporate data warehouse.

^b^VHA: Veterans Health Administration.

^c^ICD-10: International Classification of Disease-10.

^d^SCI: spinal cord injury.

^e^Not applicable.

^f^VA: Veterans Affairs.

**Table 2:** Definition of variables for aim 2b.

Variable type and name		Definition
**Exposure variable**		
	Antibiotic use	Antibiotics within 7 days of urine culture
**Outcome variables** **(within 60 days of annual evaluation encounter)**		
	Emergency visits	As recorded in CDW^a^ emergency domain
	GU^b^ complication	ICD-10^c^ codes for hematuria, urethral stricture, urethral injury, acute kidney injury, UTI^d^
	Admission for GU complication	Presence of an admission with above ICD-10 codes in CDW inpatient domain
	*Clostridium difficile* infection	Positive culture or toxin result, per CDW micro
	Repeat urine culture	Presence of urine culture in CDW microdomain
	Diagnosis of GU stone	ICD-10 codes for bladder, ureter, renal calculus
**Covariates**		
	Age	Calculated using date of birth CDW patient domain
	Gender	Male or female
	Race or ethnicity	As per VHA^e^ convention
	Neurologic level of injury	ICD-10 codes for complete and incomplete quadriplegia and paraplegia
	Bladder management strategy	Catheter prescriptions in pharmacy data

^a^CDW: corporate data warehouse.

^b^GU: genitourinary.

^c^ICD-10: International Classification of Disease-10.

^d^UTI: urinary tract infection.

^e^VHA: Veterans Health Administration.

#### Development of Provider and Nursing Intervention

For the provider intervention, the study team will conduct a series of focus groups with the SCI clinic team (providers and nurses) to receive feedback on the proposed intervention. The intervention design will be based on antibiotic stewardship principles of right drug, right dose, and right duration. The duration and number of meetings will be determined by the group and adjusted in response to the pace of progress. The components of the provider intervention will be refined through iterative design to address the potential barriers and facilitators to adoption of their use. Lab order sets will be refined to help providers not send urinalysis and urine cultures on asymptomatic patients during the annual evaluation and, if they are inadvertently sent, to prevent providers from ordering antibiotics. The current annual evaluation laboratory order set in the Houston Veterans Affairs electronic medical record includes a urinalysis and urine culture; this will be removed. The decision aid will be a pocket card that makes the ASB and UTI guidelines actionable and, thus, applicable to individual patients at the point of care. If providers state during focus groups that having a communication tool would be helpful, *talking points* will be developed on how to discuss bladder management, urine testing, and bacteriuria treatment with patients during the annual evaluation. Nursing interventions will include developing a script and symptom checklist for nurses to use when interacting with patients requesting urine testing and bacteriuria management during the annual evaluation. Although the focus of the intervention is on helping providers decide whether to use antibiotics or not, we will also provide education and material on how to choose the right drug given latest information about antibiotic resistance in organisms causing UTI in SCI.

Attending, resident, and physician extender education sessions will be incorporated into the weekly SCI lecture series. The first such meeting will introduce the intervention and underlying rationale, review the relevant ASB and UTI guidelines, and explain how and when core components such as order sets will be introduced. After this initial introductory meeting, the decision aids and other intervention materials will be given to all participants. During follow-up sessions, a case example from the annual evaluation clinic from the prior month will be discussed using the decision aid to clarify whether management was or was not compliant with ASB/UTI guidelines. This type of small-group, case-based audit and feedback was highly effective in the *Kicking CAUTI* campaign, as it retains key characteristics of effective audit and feedback, in that it is personalized (to the clinic), timely, nonpunitive, and provides the correct answer [14]. Group discussion will enable providers to ask questions about other cases they managed recently. Nursing education and audit and feedback will be done monthly during nursing in service by the study team.

#### Development of Patient Intervention

Aim 3b focuses on developing patient intervention materials. As an analogous intervention to *Kicking CAUTI* for patients does not exist, the second part of this aim will focus on ways to intervene on the patient aspect of urine testing and bacteriuria management. The intervention components will focus on patient education on objectives such as understanding neurogenic bladder and bladder management strategies after SCI and how the consequences of neurogenic bladder and/or catheterization place persons at higher risk for bacteriuria. Intervention components will include a webpage linked to the Texas Paralyzed Veterans of America (PVA) homepage with information targeting the above objectives, a paper flyer summarizing information such as that presented on the website, as well as *talking points* on how to ask a provider for more information on neurogenic bladder and bacteriuria management. We will create a video that will be housed on the PVA website, which can be readily accessed on the computers in the clinic room.

Aim 3c is to conduct a quasi-experimental pilot study of the *Test Smart, Treat Smart* intervention at the Houston VHA. For providers, the main objectives of this pilot study are to assess the following: (1) time to use or complete components of the intervention and (2) perceived burden of using intervention components. For patients, the main objectives of the pilot study are to test the following: (1) their quality of life regarding bladder management before the annual evaluation and (2) satisfaction with neurogenic bladder management and bacteriuria testing education received during the annual evaluation. A *Test Smart, Treat Smart* kick-off meeting with clinic staff will occur before the start of the intervention trial. It will also be discussed at the local PVA meeting the month before the start of the trial. The trial of the intervention will total 4 months. At the 2- and 4-month marks, providers and patients will participate in separate focus groups to provide feedback about the intervention. Patients will receive the SCI-Quality of Life-Bladder Complication assessment before the annual evaluation encounter. This is a validated instrument exploring how bladder management affects patients emotionally, and it has 6 items specifically for UTI [28]. To assess the fidelity of the intervention and prudent safety monitoring, we will compare rates of clinic visits and hospitalizations for UTI/CAUTI before the intervention, at the midpoint of the intervention, at the end of the intervention, and 2 months after the intervention.

#### Analysis for Objective 3

The qualitative responses will be coded and analyzed as described for objective 1. The SCI-Quality of Life scores will give us ideas on baseline satisfaction with bladder management to be used for future effectiveness studies. Descriptive statistics will be used to analyze the rates of the hospitalization and clinic visits for UTI/UTI for safety monitoring, and a *t* test will be used to analyze the difference in the rates of these outcomes at different time points.

### Declarations

#### Ethics Approval and Consent to Participate

This protocol has been approved by the Baylor College of Medicine institutional review board (IRB) and VHA Research and Development (H-38357).

#### Aim 1

The qualitative interviews and quantitative surveys will be performed after verbal informed consent and approved waiver of written informed consent.

#### Aim 2

The database analysis will be completed under approved waiver of informed consent.

#### Aim 3

The focus groups will be completed after verbal consent is obtained with approved waiver of written informed consent. The quasi-experimental study will be completed after verbal consent is obtained and with approved waiver of written informed consent.

## Results

This protocol received IRB and VHA Research and Development approval in July 2017, and the funding start date for the project was November 2017. The initial plan of having patients complete a Web-based survey had to be abandoned to achieve IRB approval; phone surveys are being completed instead, which has proven to be challenging. We have recently made the addition of a scripted phone message identifying who we are and why we are calling, informing potential participants that we are not solicitors. Recruitment for aim 1 is progressing; as of submission of this manuscript, 10 (67%) of the target goal of 15 provider interviews were complete, and 77 (77%) of the target goal of 100 surveys. With regard to patients, 5 (33%) of the target goal of 15 interviews were complete, and 20 (20%) of the target goal of 100 surveys had been completed. The study team plans to present the results for providers in April 2019. Database analyses for aim 2 will begin in January 2019.

## Discussion

Our previous work shows that the resources for effective antibiotic stewardship programs are more likely to be in place in VHA facilities with SCI units versus hospitals that do not [29]. Specifically, VHA facilities with SCI centers are more likely to have at least 1 full-time infectious diseases physician, an infectious diseases fellowship program, and a clinical pharmacist with formal infectious diseases training—all characteristics that have been shown to decrease antibiotic use in previous studies [30]. However, deploying these resources effectively requires a more complete understanding of the barriers and facilitators of their use, which is the purpose of this protocol.

We anticipate several challenges in carrying out the work as described in this protocol. Difficulty in scheduling time for the provider interviews and surveys due to busy clinic schedules is anticipated. The team will work individually with providers to find a convenient time for their interviews. The study team has strong contacts with national VHA SCI leadership and will enlist their help in contacting local SCI leadership in the various sites to explain our project and encourage participation. We are aware that a database study cannot provide an explanation for why a test was done or what factors went into the provider’s decision to place the order for a test or antibiotics. For this reason, the findings from our database studies of the clinical outcomes downstream from the annual urine testing will be triangulated with our qualitative results. The association we expect to find between urine tests and higher use of antibiotics, even potentially *Clostridium difficile*, will provide evidence to make our intervention acceptable to end users. In other words, if we find that potential harms result from routine urine testing in SCI, this finding will encourage people to adopt new practices. The literature supports the concept that interventions are more likely to be adopted if the evidence base is considered trustworthy and source of evidence is relevant [31].

Bacteriuria management in persons with SCI is a routine task that involves complex decision making to be handled in a guidelines-compliant manner, particularly the given conflicting guidelines on this topic. Our future work will focus on the implementation and sustainability of antibiotic stewardship interventions for bacteriuria in this high-risk population. We have developed regional and national partners on this work to increase the likelihood of widespread adoption.

## Appendix

Multimedia Appendix 1 Grant summary statement.

Multimedia Appendix 2 Funding announcement letter.

## Abbreviations

ASB: asymptomatic bacteriuria
CAUTI: catheter-associated urinary tract infection
CDW: corporate data warehouse
ICD: International Classification of Disease
IRB: institutional review board
MMAS-8: Morisky Medication Adherence Scale-8
PVA: Paralyzed Veterans of America
SCI: spinal cord injury
UTI: urinary tract infection
VHA: Veterans Health Administration
