# Supplementary material for: Determining Best Practices for Management of Bacteriuria in Spinal Cord Injury: Protocol for a Mixed-Methods Study
Source: JMIR Res Protoc. 2019 Feb 14;8(2):e12272. doi: 10.2196/12272 (PMC6393777; doi:10.2196/12272)
Supplement: Multimedia Appendix 1 [file resprot_v8i2e12272_app1.pdf]

SUMMARY STATEMENT

PROGRAM CONTACT: (Privileged Communication) *Release Date:* 03/27/2017
Revised Date:

Application Number: 1 IK2 RX002633-01

Principal Investigator

SKELTON, FELICIA

Applicant Organization: MICHAEL E DEBAKEY VA MEDICAL CENTER

Review Group: HBCU
VA-ORD Historically Black College and University Research Scientist Training Program

Meeting Date: 03/03/2017 *RFA/PA:* RX17-008
Council: MAY 2017
Requested Start: 06/01/2017

Project Title: Optimizing Bacteriuria Management in Veterans with Spinal Cord Injury

SRG Action: Impact Score:103

Human Subjects: 20-Human subjects involved - No exemption designated
Animal Subjects: 10-No live vertebrate animals involved for competing appl.

Project Year	Direct Costs Requested
1	214,270
2	242,781
3	242,803
4	247,475
5	251,629
TOTAL	1,198,958

ADMINISTRATIVE BUDGET NOTE: The budget shown is the requested budget and has not been adjusted to reflect any recommendations made by reviewers. If an award is planned, the costs will be calculated by VA Office of Research and Development (ORD) staff based on the recommendations outlined in the BUDGET COMMENT section and any relevant ORD service-specific limitations.

SUMMARY OF DISCUSSION:
The Board met in Plenary Session and reviewed the above proposal considering all internal and external reviews. This document summarizes the major points of the discussion concerning the proposed project. In any further development of this project, the investigator should consider carefully all the issues reflected in this Summary of Discussion as well as the more detailed comments in the individual critiques.

GENERAL COMMENTS:
This is an outstanding CDA2 application from Dr. Skelton which seeks to further training and research study in the area of asymptomatic bacteriuria in Veterans with spinal cord injury. Overall, the Board agrees that this is a highly qualified candidate who has a strong training plan and mentoring team supporting this project. In addition, the proposed research addresses an important area of investigation. The findings will contribute to the health and healthcare of Veterans, and the field in general, and so this application is very significant. The applicant has assembled an outstanding team of mentors and laid out an appropriate and well-crafted mentoring plan to guide her career development. The adequacy of the data to be collected and analyzed appears to be sufficient. The applicant and mentor team are well-versed in the inadequacies of existing databases and all anticipated enrollment issues have been addressed. The distribution of the roles and responsibilities across the staff are well thought out, with justification of FTE allocations. There are plans for coordinating multiple participants, tasks and sites, and the timeline appears reasonable. Notably, the investigators utilize a novel approach, the Cabana model, to understand provider barriers to using clinical practice guidelines. Importantly, the investigators have included a patient representative on the team who has helped with the development of this research project, thus ensuring that their research is relevant to the target population. This is a highly meritorious application from a well-qualified applicant with significant potential value to Veterans healthcare. No weaknesses are noted.

SUGGESTIONS:
None noted.

COMMENTS ON BUDGET:
Budget is appropriate.

DESCRIPTION (provided by applicant):
Candidate: Dr. Felicia Skelton completed medical school at Baylor College of Medicine (BCM), residency in rehabilitation medicine at the University of Washington, serving as chief resident, and clinical fellowship in spinal cord injury (SCI) medicine at BCM. She is a current health services R&D (HSR&D) post-doctoral fellow at the Center for Innovations in Quality, Effectiveness and Safety (IQuESt) at the Michael E. DeBakey VA Medical Center (MEDVAMC) in Houston, TX. She has four first-authored publications, one submitted manuscript and two pending manuscripts. She is dedicated to a career as a clinician scientist, and as a Black female, an ideal candidate for the Historically Black College or University (HBCU) Research Scientist Training Program. Career Development and Goals: During this award period Dr. Skelton will obtain the necessary epidemiologic, qualitative methods, database analysis and implementation science skills to become an independently-funded clinician scientist exploring optimal health care delivery and outcomes for Veterans with SCI. This will be achieved through completion of coursework leading to a Master's in clinical investigation from BCM, as well as other targeted educational opportunities developed by her diverse group of mentors and advisors. Environment: IQuESt and MEDVAMC are the ideal settings to complete this research, as the premiere HSR center in the Southwest and the one of the largest SCI centers in the nation, serving over 450 Veterans in the outpatient setting annually, respectively. Dr. Skelton has the support of clinical, research, administrative and patient advocacy leadership both locally and nationally for this project. Research: Bacteriuria, ether asymptomatic (ASB) or symptomatic urinary tract infection (UTI) is common in persons SCI. Current Veterans Health Administration (VHA) guidelines recommend a screening urine culture for every Veteran with SCI during their annual evaluation, even when

symptomatic, which is contrary to other national guidelines. Our preliminary data suggest that a positive urine culture (even without signs or symptoms of infection) drives antibiotic use. As the clinical outcomes of the annual exam testing have not been explored, we theorize some Veterans are receiving antibiotics unnecessarily. The negative consequences of antibiotic overuse and antibiotic resistance are well documented, and have a national and even global focus. This study will develop an intervention aimed to educate stakeholders on evidence-based management of ASB and UTI, and guide antibiotic stewardship in this high-risk population. Aim 1 will identify patient, provider, and facility factors driving bacteriuria testing and subsequent antibiotic use after the SCI annual evaluation using qualitative interviews and quantitative surveys. Aim 2 will use national VHA databases to identify the predictors of urine testing and subsequent antibiotic use during the annual examination, and compare the clinical outcomes of those who received antibiotics to those who did not. Aim 3 will use the information gathered from the previous aims to develop the “Test Smart, Treat Smart” intervention, a combination of patient and provider education and resources that will help all stakeholders have informed conversations about urine testing and antibiotic use; feasibility will be tested at a single site. The next step will be to refine the intervention and test its feasibility and effectiveness at multiple sites, with VA merit review funding, as well as guiding policy reform surrounding this common, but burdensome condition.

PUBLIC HEALTH RELEVANCE:
After a spinal cord injury (SCI), changes in how the body stores and eliminates urine often leads to bacteria growing in the bladder. If a Veteran with SCI has signs and symptoms of a urinary tract infection (UTI), their urine should be tested for these bacteria, and if bacteria are found, treated with antibiotics appropriately. If they do not have signs and symptoms of an UTI however, several treatment guidelines recommend that the urine should not be tested in the first place. Furthermore, antibiotics should not be given. Antibiotics can harm as well as help, and Veterans with SCI are very likely to receive many courses of antibiotics over their lifetime. Our study will develop a program to help providers prescribe Veterans antibiotics for bacteria in the urine only when necessary, and to educate Veterans with SCI about best practices around urine testing and antibiotic use.

CRITIQUE 1

DESCRIPTION: No comment.

CRITIQUE (Applicant, Mentor, and Training Program):

Applicant: Dr. Felicia Skelton completed medical school at Baylor College of Medicine (BCM), residency in rehabilitation medicine at the University of Washington, serving as chief resident and clinical fellowship in spinal cord injury (SCI) medicine at BCM. She is a current health services R&D (HSR&D) post-doctoral fellow at the Center for Innovations in Quality, Effectiveness and Safety (IQuESt) at the Michael E. DeBakey VA Medical Center (MEDVAMC) in Houston, TX. She has four first-authored publications, one submitted manuscript and two pending manuscripts.

Mentor/s: The mentorship committee and plan is excellent.

Training Program: The Principal Investigator (PI) is applying for a VA career development award. There is extensive institutional commitment to his development as an academic researcher. During this award period Dr. Skelton will obtain the necessary epidemiologic, qualitative methods, database analysis and implementation science skills to become an independently clinician scientist exploring health care delivery and outcomes for Veterans. This will be achieved through completion of coursework leading to a master’s degree in clinical investigation, as well as other targeted educational opportunities developed by her diverse group of mentors and advisors. The mentoring plan is excellent including didactic, research and mentoring opportunities.

CRITIQUE (Scientific Merit—Strengths, Weaknesses, & Recommendations):

Significance: Bacteriuria, ether asymptomatic (ASB) or symptomatic urinary tract infection (UTI) is common in persons spinal cord injury and presents a major issue in Veterans.

Approach: This study will develop an intervention aimed to educate stakeholders on evidence-based management of ASB and UTI, and guide antibiotic stewardship in this high-risk population. Aim 1 will identify patient, provider, and facility factors driving bacteriuria testing and subsequent antibiotic use after the spinal cord injury annual evaluation using qualitative interviews and quantitative surveys. Aim 2 will use national VHA databases to identify the predictors of urine testing and subsequent antibiotic use during the annual examination, and compare the clinical outcomes of those who received antibiotics to those who did not. Aim 3 will use the information gathered from the previous aims to develop the “Test Smart, Treat Smart” intervention, a combination of patient and provider education and resources that will help all stakeholders have informed conversations about urine testing and antibiotic use; feasibility will be tested at a single site.

Environment: Michael E. DeBakey VA Medical Center is one of the largest facilities in the national VA Medical Center system is an appropriate place. Baylor College of Medicine is also an appropriate training environment.

Feasibility: The studies are feasible and if successful findings from this study could develop policies for Veterans with spinal cord injury.

Overall Evaluation (Applicant/Mentor(s)/Training and Scientific Merit):
This is a well written proposal from a highly qualified applicant. The subject matter is relevant to diseases that plague Veterans. The training plan is excellent and the mentors are superb. The scientific merit of the application is high and the aims are accomplishable.

Ethical/Safety Issues: None noted.

OTHER CONSIDERATIONS:

Clinical Relevance: Spinal cord injury occurs in Veterans and commonly is given prophylactic antimicrobial therapy to overcome UTI and other perceived infections. There is a need to develop better policies for use of antibiotics in Veterans and important knowledge gaps must be filled before an effective antibiotic stewardship program can be implemented in this patient population.

Budget: The budget is reasonable.

Other Issues: None noted.

CRITIQUE 2

DESCRIPTION: No comment.

CRITIQUE (Applicant, Mentor, and Training Program):

Applicant: Dr. Skelton is fellowship trained in SCI clinical care, and is a postdoctoral fellow in health services research, and is enrolled as a Masters student in clinical investigation.
Relevant work is cited and Dr. Skelton’s preliminary data over 2 years indicates that this practice has resulted in unnecessary treatment in 35% of cases classified as asymptomatic bacteriuria in their

facility. This is potentially a huge problem for generation of antibiotic resistant strains in patients who are chronically colonized and asymptomatic. She is proposing to extend the study of this practice nationally to gain an understanding of what the factors are that drive antibiotic over-usage.

Mentor/s: The mentorship team is outstanding.

Training Program: The mentoring team is based on her scientific objectives and training gaps. Co-mentor Dr. Trautner is an MD PhD with successful mentoring experience (mentees have continued to perform independently funded research) and an expert in catheter-associated UTI. Dr. Trautner is also a staff physician at MEDVAMC and Associate professor at BCM. Co-mentor Dr. Chui is a professor of Pharmacy at Texas Southern University and a WOC at BCM and MEDVAMC. She has extensive experience with VA databases. She will help develop the qualitative interviews for patients, provide guidance on VHA data sources, and make sure medication adherence themes are captured. The advisory committee includes DR. Holmes MD, who is the SCI care line executive for MEDVAMC, the SCI Fellowship director at BCM and has extensive experience as a site PI for VA cooperative studies. Dr. Martin PhD is a medical anthropologist who will guide the qualitative methodology and statistical analysis. Dr. Evans MPH PhD is an epidemiologist VA Office of Public Health National Center for Occupational Health and Infection Control (COHIC) and an associate professor at the Center for Healthcare Studies and Department of Preventive Medicine in the Feinberg School of Medicine at Northwestern University. She is the former director of the VA SCI Quality Research Enhancement Initiative (QUERI), and is the current co-PI of a newly awarded QUERI (similar to National Institutes of Health (NIH) U-grant) focused on antimicrobial stewardship. Consultants include Anne Robinson, the President of the Texas Chapter of the PVA, who will provide patient perspective and access to SCI patient focus groups, and Dr. McDeavitt MD is Chair of the Department at BCM, and will ensure the protected research time. In short, this is an all-star team that should ensure the success of Dr. Skelton.

CRITIQUE (Scientific Merit—Strengths, Weaknesses, & Recommendations):

Significance: The proposed work is highly significant for Veterans and patients with spinal cord injury. Patients with SCI have a prevalence of 30-90% asymptomatic bacteriuria, dependent upon bladder management strategy. This compares to pre-menopausal women at 5%. Asymptomatic bacteriuria is not recommended for either screening or treatment in SCI patients by the Infectious Disease Society of America. The VHA guideline, however, recommends screening at annual examinations regardless of the presence or absence of symptoms. The proposed research is important for the health care delivery and outcomes in VA, nationwide and beyond because it addresses both dangers of over-treatment and unnecessary testing that increases health care costs. Everyone is aware of the dangers of antibiotic over-usage, and thus this research is of high importance and impact because the ultimate goal is to correct the existing VHA guidelines if she finds that they are potentially causing more harm than good.

Approach: In SA1, they will identify contextual factors influencing provider and patient understanding and beliefs about urine testing and treatment at the annual examination, using quantitative and qualitative methods. In SA2, they will identify the evidence regarding patient, provider and facility predictors of urine testing and subsequent antibiotic use, as well as compare the clinical outcomes of those who received antibiotics with the outcomes of those who did not. In SA3, they will use information gained from the above aims and previously successful antibiotic stewardship initiatives to intervene, by providing evidence-based bacteriuria management through education and resources for patients and providers. The addition of a medical anthropologist to assess how organizational culture affects medical practice in SCI clinics and a patient representative to help in constructing the intervention on the team is a big plus. The conceptual or clinical framework, design, methods, and analyses are adequately developed, well-integrated, well-reasoned, and appear to be appropriate to the aims of the project. They address potential pitfalls and alternative approaches.

Environment: The facilities and resources are exemplary and will support the successful execution of the proposed studies.

Feasibility: The planned studies are highly feasible and the group assembled is uniquely qualified to carry them out to completion.

Overall Evaluation (Applicant/Mentor(s)/Training and Scientific Merit):
This is a well thought out project with a strong team addressing an important issue impacting the care of SCI patients. The applicant's qualifications are outstanding and represent a high degree of future potential. The training plan is well-conceived and the mentors are of the highest quality.

Ethical/Safety Issues: None.

OTHER CONSIDERATIONS:

This study has very apparent clinical value. The benefit to the Veterans with spinal cord injury will be reduction in risk of developing antibiotic resistant bacteriuria, reduced treatment load, greater understanding of asymptomatic bacteriuria and chronic colonization, which should all lead to an improved Quality of Life. An additional major benefit to VHA would be reduction of costs due to testing.

Budget: The budget is appropriate.

Other Issues: None

CRITIQUE 3

DESCRIPTION: No comment.

CRITIQUE (Applicant, Mentor, and Training Program):

Applicant: Dr. Felicia Skelton completed medical school at Baylor College of Medicine, residency in rehabilitation medicine at the University of Washington, serving as chief resident, and clinical fellowship in spinal cord injury medicine back at Baylor. She is a current health services R&D post-doctoral fellow at the Center for Innovations in Quality, Effectiveness and Safety at the Michael E. DeBakey VA Medical Center in Houston, TX. She has four first-authored publications, one submitted manuscript and two pending manuscripts. She would like to become a strong clinician scientist.

Mentor/s: The mentorship team is excellent and well-suited to guide the career development of Dr. Skelton.

Training Program: The training plan is explained well. In addition, during this award period Dr. Skelton will obtain the necessary epidemiologic, qualitative methods, database analysis and implementation science skills to become an independently-funded clinician scientist exploring optimal health care delivery and outcomes for Veterans with spinal cord injury. This will be achieved through completion of coursework leading to a master's degree in clinical investigation, as well as other targeted educational opportunities developed by her diverse group of mentors and advisors.

CRITIQUE (Scientific Merit—Strengths, Weaknesses, & Recommendations):

Significance: The proposed work is has high potential significance. While bacteriuria after SCI is common, and there is an urgency regarding the fact that this population needs antimicrobial stewardship, there are still important knowledge gaps. The investigators identify these gaps as:

- A need to understand what SCI patients believe, want, and expect in regards to having their urine tested annually, and being prescribed, and adhering to prescribed antibiotics.
- A need to understand providers' concerns and rationale behind urine testing in the absence of symptoms.
- A need to understand the outcome of these testing and treatment decisions on a national scale.

The proposed work seeks to acquire important information to address these gaps. Therefore, what the proposed research would address is important to the health care delivery and outcomes in VA, nationwide and beyond. Furthermore, the investigators utilize a novel approach, the Cabana model, to understand provider barriers to using clinical practice guidelines. In addition, the investigators have included a patient representative on the team who has helped with the development of this research project, thus ensuring that their research is relevant to the target population. The VHA is the premiere system for caring for patients with SCI and employs physiatrists as primary care physicians for persons with SCI. VHA standards of care for persons with SCI includes the annual examination, which includes a comprehensive medical evaluation, including laboratory and radiologic testing, as well as functional and psychosocial evaluations. Currently, these standards recommend a urine culture and urinalysis as part of this evaluation, regardless of the presence of urinary symptoms. The annual examination is a clearly defined encounter of care for Veterans with SCI. Therefore, identifying key stakeholders to discuss barriers and facilitators for bacteriuria management will be clear. Information from this study can inform VHA policy, which will standardize care for Veterans with SCI nationwide.

Approach: This study will develop an intervention aimed to educate stakeholders on evidence-based management of ASB and UTI, and guide antibiotic stewardship in this high-risk population. Aim 1 will identify patient, provider, and facility factors driving bacteriuria testing and subsequent antibiotic use after the SCI annual evaluation using qualitative interviews and quantitative surveys. Aim 2 will use national VHA databases to identify the predictors of urine testing and subsequent antibiotic use during the annual examination, and compare the clinical outcomes of those who received antibiotics to those who did not. Aim 3 will use the information gathered from the previous aims to develop the "Test Smart, Treat Smart" intervention, a combination of patient and provider education and resources that will help all stakeholders have informed conversations about urine testing and antibiotic use; feasibility will be tested at a single site. The next step will be to refine the intervention and test its feasibility and effectiveness at multiple sites, with VA merit review funding, as well as guiding policy reform surrounding this common, but burdensome condition.

Environment: The environment is ideal to perform this study. Facilities and resources are more than adequate. The Center for Innovations in Quality, Effectiveness and Safety at the Michael E. DeBakey VA Medical Center in Houston, TX, looks like the ideal setting to complete this research, serving over 450 Veterans in the outpatient setting annually, respectively. Dr. Skelton has the support of clinical, research, administrative and patient advocacy leadership both locally and nationally for this project.

Feasibility: The study as stated is highly feasible.

Overall Evaluation (Applicant/Mentor(s)/Training and Scientific Merit):
Overall, this is a strong candidate and team, with a well laid out project, exceptional facilities and environment.

Ethical/Safety Issues: None.

OTHER CONSIDERATIONS

Budget: The budget is acceptable.

Other Issues: None.

MEETING ROSTER
VA-ORD Historically Black College and University Research Scientist Training Program
Rehabilitation Research and Development Parent IRG
Office of Research & Development

HBCU
03/03/2017

CHAIRPERSON(S)

STERGIOU, NICK, PHD
DISTINGUISHED COMMUNITY RESEARCH CHAIR AND PROFESSOR
CHAIR, DEPARTMENT OF BIOMECHANICS
UNIVERSITY OF NEBRASKA AT OMAHA
UNIVERSITY OF NEBRASKA MEDICAL CENTER
OMAHA, NE 68182

MEMBERS

AGOSTON, DENES V., PHD, MD *
PROFESSOR
ANATOMY, PHYSIOLOGY AND GENETICS
F. EDWARD HEBERT SCHOOL OF MEDICINE
UNIFORMED SERVICES UNIV OF THE HEALTH SCIENCES
BETHESDA, MD 20814

BAI, GUANG, MD, PHD *
ASSISTANT PROFESSOR
DEPARTMENT OF NEURAL AND PAIN SCIENCES
UNIVERSITY OF MARYLAND SCHOOL OF DENISTRY
BALTIMORE, MD 21201

BRENNER, LISA A, PHD *
DIRECTOR
VISN 19 MIRECC, DENVER VA MEDICAL CENTER
PROFESSOR
DEPARTMENT OF PSYCHIATRY
UNIVERSITY OF COLORADO SCHOOL OF MEDICINE
DENVER, CO 80220

BROWN, MILTON L., PHD, MD *
PROFESSOR
DEPARTMENT OF ONCOLOGY
GEORGETOWN UNIVERSITY MEDICAL SCHOOL
WASHINGTON, DC 20057

CARDOZO, CHRISTOPHER, MD, PHD *
STAFF PHYSICIAN
BRONX VA MEDICAL CENTER
CENTER OF EXCELLENCE
FOR THE MEDICAL CONSEQUENCES OF SCI
BRONX, NY 10468

CONTI, ALANA C., PHD *
HEALTH SCIENCE SPECIALIST
JOHN D. DINGELL VA MEDICAL CENTER
ASSISTANT PROFESSOR
DEPARTMENT OF NEUROSURGERY
WAYNE STATE UNIV SCHOOL OF MEDICINE
DETROIT, MI 48236

COOPER, RORY A., PHD *
DIRECTOR
VA PITTSBURGH HEALTH CARE SYSTEM
PROF, DEPT OF REHAB SCIENCE AND TECH
SCHOOL OF HLTH AND REHAB SCIENCES
UNIVERSITY OF PITTSBURGH
PITTSBURGH, PA 15206

CROSSON, BRUCE A., PHD *
RESEARCH ASSOCIATE
ATLANTA VAMC
PROF, CTR FOR VISUAL & NEUROCOGNITIVE REHAB
EMORY UNIVERSITY
ATLANTA, GA 30033

DALY, JANIS J., PHD *
DIRECTOR
BRAIN REHABILITATION RESEARCH COE
N FLORIDA/S GEORGIA VETERANS HEALTH SYSTEM
PROFESSOR, DEPARTMENT OF NEUROLOGY
UNIVERSITY OF FLORIDA
GAINESVILLE, FL 32608

DZIRASA, KAFUI, PHD, MD *

ELDER, GREGORY A., MD *
PRACTICE CHIEF
JAMES J. PETERS VA MEDICAL CENTER (BRONX)
ASSOCIATE PROFESSOR, DEPT OF PSYCHIATRY
MOUNT SINAI SCHOOL OF MEDICINE
NEW YORK, NY 10029

FRASER, MATTHEW O, PHD *
ASSOCIATE PROFESSOR
DEPARTMENT OF UROLOGY
DUKE UNIVERSITY
DURHAM, NC 27713

GORGEY, ASHRAF, PHD *
PHYSICAL THERAPIST
SPINAL CORD INJURY & DISORDERS SERVICE
HUNTER HOLMES MCGUIRE VA MEDICAL CENTER
ASSISTANT PROFESSOR, DEPT OF PM&R
VIRGINIA COMMONWEALTH UNIVERSITY
RICHMOND, VA 23249

GUSTAFSON, KENNETH J., PHD *
ASSOCIATE DIRECTOR
CLEVELAND VA MEDICAL CENTER
CLEVELAND FES CENTER
ASSOC PROF, DEPT OF BIOMEDICAL ENG & UROLOGY
CASE WESTERN RESERVE UNIVERSITY
CLEVELAND, OH 44106

HARRIS-LOVE, MICHAEL, DSC *
DOCTOR, GERIATRICS - RESEARCH
GERIATRICS AND EXTENDED CARE SERVICE
WASHINGTON DC VA MEDICAL CENTER
WASHINGTON, DC 20422

HUDSON, TAMARO SYTON, PHD *
ASSISTANT PROFESSOR
HEALTH SCIENCES
HOWARD UNIVERSITY COLLEGE OF MEDICINE
WASHINGTON, DC 20060

HUSAIN, SHAHID, PHD *
ASSOCIATE PROFESSOR
DEPARTMENT OF OPHTHALMOLOGY
STORM EYE INSTITUTE
CHARLESTON, SC 29425

KARTJE, GWENDOLYN LOUISE, MD, PHD *
PROFESSOR
DEPARTMENT OF NEUROLOGY
STRITCH SCHOOL OF MEDICINE
LOYOLA UNIVERSITY OF CHICAGO
MAYWOOD, IL 60153

KOU, ZHIFENG, PHD *
ASSISTANT PROFESSOR
DEPARTMENT OF BIOMEDICAL ENGINEERING AND
RADIOLOGY
WAYNE STATE UNIVERSITY
DETROIT, MI 48202

LYNCH, CONOR C, PHD *
ASSOCIATE PROFESSOR
ASSOCIATE MEMEBER
TUMOR BIOLOGY DEPARTMENT
H. LEE MOFFITT CANCER CENTER AND RES. INSTITUTE
TAMPA, FL 33612

MEEK, SANFORD GEORGE, PHD *
ASSOCIATE PROFESSOR
UNIVERSITY OF UTAH
DEPARTMENT OF MECHANICAL ENGINEERING
SALT LAKE CITY, UT 84112

MEI, LIN, MD, PHD *
PROFESSOR AND CHAIR
DEPARTMENT OF NEUROSCIENCE &
REGENERATIVE MEDICINE
MEDICAL COLLEGE OF GEORGIA
AUGUSTA UNIVERSITY
AUGUSTA, GA 30912

MIN, LILLIAN CHIANG, MD *
ASSISTANT PROFESSOR
300 N INGALLS BLDG ROOM 966
ANN ARBOR, MI 48103

YATES, CLAYTON, PHD *
ASSISTANT PROFESSOR
DEPARTMENT OF BIOLOGY
CENTER FOR CANCER RESEARCH
CARVER RESEARCH FOUNDATION
TUSKEGEE UNIVERSITY
TUSKEGEE, AL 36088

SCIENTIFIC REVIEW OFFICER

CUNNINGHAM, TSHAKA, PHD
SCIENTIFIC PROGRAM MANAGER
DEPARTMENT OF VETERANS AFFAIRS
OFFICE OF RESEARCH AND DEVELOPMENT
REHABILITATION RESEARCH AND DEVELOPMENT SERVICE
WASHINGTON, DC 20420

* Temporary Member. For grant applications, temporary members may participate in the entire meeting or may review only selected applications as needed.

Consultants are required to absent themselves from the room during the review of any application if their presence would constitute or appear to constitute a conflict of interest.
