# Supplementary material for: Determining Best Practices for Management of Bacteriuria in Spinal Cord Injury: Protocol for a Mixed-Methods Study
Source: JMIR Res Protoc. 2019 Feb 14;8(2):e12272. doi: 10.2196/12272 (PMC6393777; doi:10.2196/12272)
Supplement: Multimedia Appendix 2 [file resprot_v8i2e12272_app2.pdf]

Application Number: 1IK2HX002484-01

Principal Investigator(s):
Felicia Skelton

Project Title: Optimizing Bacteriuria Management in Veterans with Spinal Cord Injury

Kinsel, Paula
Michael E. DeBakey VA Medical Center
2002 Holcombe Blvd. (151)
Houston, TX 770304298

Budget Period: 11/01/2017 – 10/31/2018
Project Period: 11/01/2017 – 10/31/2022

We are pleased to inform you that funding in the amount of $214,270 (see “Proposed Award Data” in Section I) is being awarded to support the above named project. Funds will be provided by fiscal year according to the award calculation and the budget start date. The funding amounts cited in this Notice of Award may not match the allocations provided to your medical center because funds are provided to VA sites by fiscal year. The VA fiscal year starts on October 1st and ends on September 30th each year. Minor changes to the budget will not be reflected here but can be found in the “Pink Sheet” generated from the VA RAFT program. Contact the VA Research Office at your medical center to obtain a copy of your “Pink Sheet” which will provide you with fiscal year funding amounts and will incorporate changes to the budget.

Please refer to Section III for Terms and Conditions of the award.

If you have questions about this award, please contact the individual(s) referenced in Section II below.

SECTION I – PROPOSED AWARD DATA – 1IK2HX002484-01

SUMMARY OF PROPOSED TOTALS FOR ALL YEARS	
**YR**	**AWARD**
1	$214,270
2	$242,781
3	$242,803
4	$247,475
5	$251,629

Recommended future year total cost support is subject to the availability of funds and satisfactory progress of the project.

SECTION II – CONTACT INFORMATION – 1IK2HX002484-01

The Program Officer (PO) named below is responsible for the scientific, programmatic and technical aspects of this project.

PO: Robert Small
Email: robert.small@va.gov Phone: 202-443-5743

SECTION III – TERMS AND CONDITIONS – 1IK2HX002484-01

This award is based on the application submitted to and approved by VA-ORD and is subject to the terms and conditions incorporated either directly or by reference in the following:

a. Award recipients must adhere to VA research policies including VA requirements for progress reports (RPPRs); final reports; research publications; public access to data; intellectual property; and clinical trial registration and reporting required by clinicaltrials.gov.
b. All awards are dependent upon the availability of funds.
c. Awards should be carried out according to the plan presented in the approved application. Any significant changes in project duration, budget, aims, methods, site(s) or PI must be approved by Health Services Research & Development. Project modifications may be requested using the ORD Project Modification Form located on the Office of Research & Development website at http://www.research.va.gov/resources/policies/default.cfm.

Addendum to Section III – Terms and Conditions

Budget Information

Support beyond the current fiscal year is contingent upon the availability of HSR&D funds and satisfactory progress of the research. The project budget will be transmitted electronically to the Research and Development Computer Center (RDCC) office for distribution to the field.

- **Unfilled to-be-named (TBN) positions.** When funds are required for previously unfilled positions, please e-mail Mary Jones at Mary.Jones@va.gov with a copy to the Scientific Program Manager. The following information is needed: amount of budgeted funds needed and number of people to be hired.

For questions regarding the distribution of project funds, your administrative officer should contact Mary Jones at 202-443-5628 or Mary.Jones@va.gov.

Reporting Requirements

1. HSR&D requires three types of regular reports for every research project: annual progress report; copies of all publications based on the HSR&D-funded work; and a final report. In addition, ORD (and HSR&D) requires quad charts to be submitted for all funded studies, with the initial abstract as well as with the final report. Approval of future HSR&D funding is contingent on the investigator's adherence to these critical requirements. For additional information and details regarding investigator reporting requirements, please consult your local R&D office.

   a. Annual Progress Report. HSR&D has an annual reporting requirement for all its funded studies. Reporting will occur within the VA-Office of Research and Development (ORD) Research Performance Progress Report (RPPR) function in NIH eRA Commons. Please collaborate with your local research office to complete the RPPR via NIH eRA Commons (http://grants.nih.gov/grants/rppr/index.htm). VA-ORD Instructions for preparing and submitting an RPPR are available at http://www.research.va.gov/resources/RPPR.cfm. The RPPR is due 45 days before the next budget period start date for each funded study on an annual basis.

   b. Publication Transmittal. Investigators are required to promptly notify HSR&D of all publications resulting from HSR&D-funded research. Submit your notification as soon as it is accepted for publication, by following the steps below:
   - Go to the PubTracker Website by copying and pasting the following URL into your browser: http://vaww.pubtracker.research.va.gov/PubTracker/default.cfm (Access restricted to VA Intranet using Internet Explorer)
   - Select the appropriate submission type from the "New Pre-pub Notification" and fill in the form (Be sure to upload a copy of the complete accepted article or presentation abstract).

   c. Final Report. A Final Report, (conforming to current HSR&D instructions) and quad chart are required at the conclusion of the funding period. Final Report instructions are available on HSR&D Website: http://www.hsrd.research.va.gov/funding/final_reports.cfm.

VA Acknowledgement

Each publication, press release or other document that cites results from VA-supported research must include an acknowledgment of VA support using the eRA application number, such as "The project described was supported by (type of award, e.g., Merit Review, Career Development Award, Pilot Project) Award Number I01 HX002067 from the United States (U.S.) Department of Veterans Affairs Health Services Research & Development Service of the VA Office of Research and Development." When the work was solely funded by VA, authors must list their VA affiliation first. When the author also holds a faculty appointment, the academic title and school also may be acknowledged. All publications should include a disclaimer similar to this statement: "The views expressed in this article are those of the author(s) and do not necessarily represent the views of the Department of Veterans Affairs."

Other Communications

If, while conducting this study, you encounter any research barriers that HSR&D should be notified about and/or may assist in resolving, they should be forwarded to HSR&D by the COIN AO or Research AO via the following link: http://vaww.hsrd.research.va.gov/research-barriers/. Please include the project number (above) in any communication concerning this project. Please be reminded that all communication regarding this project should go through the local research office. The principal investigator is responsible for relating all communications from VA Central Office to any co-investigators, as necessary.

Fiduciary Responsibility

It is the fiduciary responsibility of each PI, working with the local ACOS/R&D staff, to monitor the status of funding expenditures and ensure that funds are spent as described on the project budget. Funds allocated in a given fiscal year must be spent by the end of that fiscal year. In rare circumstances, CO will consider written requests to "pull back" funds but cannot guarantee that they will be returned in ensuing fiscal years. Requests to "pull back" funds must be submitted by the AO at the local R&D office to CO. Requests to "pull back" funds for return in future fiscal years are more likely to be approved if they are made early in the fiscal year or if CO has been advised of a potential problem that becomes real. Requests made after June 30th are not likely to be approved, especially if CO has not received communication of a potential problem. **ANY FUNDS THAT ARE NOT SPENT WILL BE WITHDRAWN.** It is the responsibility of the PI to notify the local R&D office if a move to another VA is anticipated or if you expect to leave the VA.

To help you to meet this fiduciary obligation, we offer the following recommendations:

- Request a monthly expenditure report from your local ACOS/R&D office and review it to ensure that all charges are accurate and approximately 1/12 of your proposed budget is spent monthly
- Request notification when a Transfer Disbursing Authority (TDA) for your project has been received on station
- Communicate with your local R&D office **as soon as you are aware** of a personnel problem such as:
    - Inability to spend all of the funds allocated to you for salary due to a project member leaving or out on extended leave (sick leave or family leave)
    - Inability to spend all of the funds allocated to you for salary due to a delay in recruiting or hiring project staff
- Communicate with your local R&D office if you will be unable to recruit an adequate number of subjects (and this will delay completion of the study)

- Communicate with your local R&D office if you are unable to obtain access to datasets that are necessary for the completion of your study or if there are problems with the data sets which will delay completion of the study
- Communicate with your local R&D office if there are problems spending IT funds. Check the status of IT requests to ensure that they have been entered into the request and approval system
- Ask your local ACOS/R&D office to include you on the “cc” line of any communication of problems/issues to CO to ensure that the communication you have initiated is transmitted.

If the project is a multi-site study, it is the responsibility of the PI to monitor expenditures at all sites, and communicate potential problems to his/her own local R&D office.

Once HSR&D funding is initiated, investigators must obtain formal approval from the Director, HSR&D, for any significant change in the approved project research plan, objectives, methods, budget, time, key personnel, or site(s). **Approval of requests for modifications will be based on compelling justification, e.g., for situations beyond the control of the Principal Investigator that affect completion of a project as approved.**

All requests for project modifications must be submitted by the medical center Director, through the ACOS for R&D and the Center Director (if applicable) to the Director, HSR&D. To permit careful review, all modification requests must be submitted as soon as the need becomes apparent and, in all cases, at least 3 months prior to the effective date of the proposed change. Justification for the requested modification must be clear, detailed, and contain appropriate supporting documentation, including revised budgets, timelines, letters of support, etc., as applicable. If additional information is required, the Principal Investigator will have 30 days from the date of communication to respond to the request; if this deadline is not met, the request may be disapproved. Unusual or extraordinary circumstances that preclude a response by the deadline must be discussed with the Scientific Program Manager for the project. Current instructions on required format and content for requesting Project Modifications can be found at HSR&D’s website: http://www.hsrd.research.va.gov.

Department of Veterans Affairs	MERIT REVIEW BOARD SUMMARY STATEMENT

1. PROJECT ID	2. APPLICATION NO	3. REVIEW GROUP	4. REVIEW DATE	5. FACILITY NO
CDA 17-170	824-CD-CI-34111	Career	W 2017/05	580

6. LOCATION HEALTH CARE FACILITY (VAMC, OPC, CITY, STATE)	7. CID#	8. DATE OF LAST M.R.
Houston, TX	FSKLETON	

9. INVESTIGATOR (Last Name, First Name, M.I.)	DEGREE(S)	TELEPHONE NO.
Skelton, Felicia	M.D.	713-791-1414 10218

Remarks: Hold funding for TBN $40, 829; advise when hired.

10. Project Title	Start Date:	End Date:
Optimizing Bacteriuria Management in Veterans with Spinal Cord Injury	Nov 1, 2017	Oct 31, 2022

11. AMOUNT REQUESTED EACH YEAR

1st	2nd	3rd	4th	5th	TOTAL

12. VA EMPLOYMENT

- [x] Full Time
- [ ] Part Time
- [ ] Consulting
- [ ] Contract
- [ ] WOC

13. VA SALARY SOURCE

- [ ] Research CC103
- [ ] Research CC104
- [ ] Research CC105
- [ ] Research CC110
- [ ] Career Dev. CC108
- [ ] Medical Research (821)
- [ ] HSR&D (824)
- [ ] Rehab R&D (822)
- [ ] Coop Studies (825)
- [ ] VA Other Than R&D

14. TYPE PROGRAM

- [ ] New
- [ ] Ongoing
- [ ] Supplement
- [ ] Type II
- [ ] NO. Projects In Program

15. PROGRAM: 824 Health Services

16. Primary Research Program Area	Primary Specialty Area
Health Services	Physical Medicine & Rehabilitation

17a. VA Hospital Service	18a. Academic Rank, Affiliation
Spinal Cord Injury	Assistant Professor, Baylor College of Medicine

17b. VA Hospital Section	18b. Department & Section
	Physical Medicine and Rehabilitation,

Recomendation	Scores	Duration	Start Date	End Date
			11/01/2017	10/31/2022

134 HSR&D

FUNDS NOT SUBJECT TO PRIORITY REDUCTION					
FY	Salary	Equipment	Travel	All Other	Total
2018	$ 167,131	$ 0	$ 0	$ 6,175	$ 173,306
2019	$ 231,352	$ 0	$ 0	$ 15,337	$ 246,689
2020	$ 235,935	$ 0	$ 0	$ 11,252	$ 247,187
2021	$ 240,601	$ 0	$ 0	$ 11,162	$ 251,763
2022	$ 245,368	$ 0	$ 0	$ 11,162	$ 256,530
2023	$ 18,906	$ 0	$ 0	$ 0	$ 18,906
